# Conformation-selective tau monoclonal antibodies inhibit tau pathology in primary neurons and a mouse model of Alzheimer’s disease

**DOI:** 10.1186/s13024-020-00404-5

**Published:** 2020-11-04

**Authors:** Garrett S. Gibbons, Soo-Jung Kim, Qihui Wu, Dawn M. Riddle, Susan N. Leight, Lakshmi Changolkar, Hong Xu, Emily S. Meymand, Mia O’Reilly, Bin Zhang, Kurt R. Brunden, John Q. Trojanowski, Virginia M. Y. Lee

**Affiliations:** Department of Pathology and Laboratory Medicine, Institute on Aging and Center for Neurodegenerative Disease Research, University of Pennsylvania School of Medicine, 3600 Spruce St. 3 Maloney, Philadelphia, PA 19104 USA

**Keywords:** Tau, Immunotherapy, Alzheimer’s disease, Tauopathies, Monoclonal antibodies

## Abstract

**Background:**

The spread of tau pathology in Alzheimer’s disease (AD) is mediated by cell-to-cell transmission of pathological tau seeds released from neurons that, upon internalization by recipient neurons, template the misfolding of naïve cellular tau, thereby propagating fibrillization. We hypothesize that anti-tau monoclonal antibodies (mAbs) that selectively bind to pathological tau seeds will inhibit propagation of tau aggregates and reduce the spread of tau pathology in vivo.

**Methods:**

We inoculated mice with human AD brain-derived extracts containing tau paired helical filaments (AD-tau) and identified two novel mAbs, DMR7 and SKT82, that selectively bind to a misfolded pathological conformation of tau relative to recombinant tau monomer. To evaluate the effects of these mAbs on the spread of pathological tau in vivo*,* 5xFAD mice harboring significant brain Aβ plaque burden were unilaterally injected with AD-tau in the hippocampus, to initiate the formation of neuritic plaque (NP) tau pathology, and were treated weekly with intraperitoneal (i.p.) injections of DMR7, SKT82, or IgG isotype control mAbs.

**Results:**

DMR7 and SKT82 bind epitopes comprised of the proline-rich domain and c-terminal region of tau and binding is reduced upon disruption of the pathological conformation of AD-tau by chemical and thermal denaturation. We found that both DMR7 and SKT82 immunoprecipitate pathological tau and significantly reduce the seeding of cellular tau aggregates induced by AD-tau in primary neurons by 60.5 + 13.8% and 82.2 + 8.3%, respectively, compared to IgG control. To investigate the mechanism of mAb inhibition, we generated pH-sensitive fluorophore-labeled recombinant tau fibrils seeded by AD-tau to track internalization of tau seeds and demonstrate that the conformation-selective tau mAbs inhibit the internalization of tau seeds. DMR7 and SKT82 treatment reduced hyperphosphorylated NP tau as measured with AT8 immunohistochemistry (IHC) staining, but did not achieve statistical significance in the contralateral cortex and SKT82 significantly reduced tau pathology in the ipsilateral hippocampus by 24.2%; *p* = 0.044.

**Conclusions:**

These findings demonstrate that conformation-selective tau mAbs, DMR7 and SKT82, inhibit tau pathology in primary neurons by preventing the uptake of tau seeds and reduce tau pathology in vivo*,* providing potential novel therapeutic candidates for the treatment of AD.

**Supplementary information:**

**Supplementary information** accompanies this paper at 10.1186/s13024-020-00404-5.

## Background

The neuropathological hallmarks of AD consist of extracellular amyloid-beta (Aβ) plaques and intraneuronal aggregates of tau protein associated with neuritic plaques (NPs), neuropil threads (NTs), and neurofibrillary tangles (NFTs) [1, 2]. Tau is a microtubule-associated protein expressed as six differentially-spliced isoforms containing either 0, 1, or 2 N-terminal acidic exons and 3 or 4 microtubule-binding repeats (MTBRs) [3]. The typically unstructured tau protein can adopt a misfolded beta-sheet conformation that aggregates into fibrils with a filament core comprised of the MTBRs and folding that enables contact of the N-terminus with the core domains to form paired helical filaments (PHFs) that assemble into NFTs [4–6]. Accumulations of tau protein closely correlate with cognitive decline and neuron death in AD patients more so than the presence of Aβ plaques [7, 8]. Although there are no mutations in the gene encoding tau protein associated with AD, mutation of the tau (*MAPT)* gene results in Frontotemporal Dementia with Parkinsonism linked to chromosome 17 (FTDP-17) [9]. Therefore, tau plays a central role in the neurodegenerative disease process and presents an attractive target for therapeutic intervention in AD and related tauopathies.

Tau aggregates propagate throughout the brain in a stereotypical spatiotemporal pattern that progresses with disease severity, beginning in the locus coeruleus and transentorhinal cortex, followed by the hippocampus and neocortex, and reaching the visual cortex at the latest disease stages [10]. Growing evidence suggests this process is mediated by cell-to-cell transmission of pathological tau seeds from a neuron containing fibrillar tau species to a normal recipient neuron [11]. Release of monomeric tau and polymeric misfolded tau from neurons may result from cell death or neuronal activity, generating both free extracellular tau and a small percentage of vesicle encapsulated tau [12–16]. Once taken up into a recipient neuron, pathological tau seeds act as a template to recruit native cellular tau into newly formed oligomers and fibrils [17–19]. Mouse models have demonstrated that intracerebral injection of either synthetic tau preformed fibrils (PFFs) or human AD-brain-derived pathological tau (AD-tau) can instigate tau pathology in regions of the brain distant from the injection site in either transgenic (Tg) mice expressing mutant human tau or non-transgenic wildtype (WT) mice [20–24]. Together, these findings provide impetus for the development of anti-tau antibodies as immunotherapeutics for AD based on the hypothesis that antibody binding to extracellular tau will prevent spread of pathological tau aggregates throughout the brain.

Several tau antibodies have been evaluated in preclinical models of AD, and these antibodies have targeted linear epitopes in the tau N-terminus [25–29], central domain [30], MTBRs [31, 32] or specific phosphorylation sites such as pThr231 [33], pSer396 [33, 34], pSer396/pSer404 [35, 36], pSer404 [37], pSer413 [34], or pSer422 [38]. A few antibodies reported to selectively bind pathological tau oligomers [39–41] have also been evaluated in vivo in addition to the conformation-selective antibody MC1 [36, 42, 43]. A handful of passive and active tau immunotherapy approaches have progressed into phase I and phase II clinical trials, as reviewed elsewhere [44]. Numerous mouse models of AD pathogenesis have been utilized to investigate the efficacy of tau antibodies for the treatment of AD, but few of these models recapitulated the concurrent tau and Aβ pathology observed in AD brains. Our laboratory recently reported that intracerebral injection of AD-tau into 5xFAD, mice that develop abundant Aβ plaques, results in the seeded aggregation of endogenous mouse tau within plaque-associated dystrophic neurites to yield NP tau, with later emergence of NFT-like pathology [45]. This model provides a novel platform to evaluate the efficacy of tau antibodies to inhibit the seeding and transmission of pathological tau aggregates in the context of Aβ neuropathological lesions. Utilizing our novel mouse model of AD pathologies, we hypothesize that conformation-selective tau mAbs will inhibit the cell-to-cell transmission of pathological tau seeds after initial intracerebral seeding with AD-tau. As a low proportion of peripherally administered IgG crosses the blood brain barrier [46], the selective targeting of pathological tau with conformation-selective antibodies will reduce binding to non-pathological tau species, whereas high-affinity linear epitope tau antibodies may be sequestered by extracellular soluble tau present in the brain interstitial fluid. We describe here the characterization of our conformation-selective tau antibodies and demonstrate that they are effective at inhibiting the formation of tau pathology in both in vitro and in vivo models of disease.

## Materials and methods

### Extraction of AD-tau

Postmortem human brain tissue from neuropathologically confirmed AD cases were used for extraction of AD-tau with approval of the University of Pennsylvania Institutional Review Board with informed consent from patients or their families as previously described [21]. Briefly, gray matter from frontal cortex was homogenized in high salt buffer (10 mM Tris-HCl pH 7.4, 800 mM NaCl, 1 mM EDTA, 2 mM DTT, with a protease inhibitor cocktail and PMSF as well as 0.1% sarkosyl and 10% sucrose) in a dounce homogenizer in nine volumes of buffer per gram tissue. This was followed by centrifugation at 10,000 g for 10 min at 4 °C. Sarkosyl was added to the pooled supernatant up to 1% and rotated 1 h at room temperature (RT) then centrifuged at 300,000 g for 1 h at 4 °C. The sarkosyl-insoluble pellet, containing pathological tau, was washed in PBS at 100 μL/g gray matter, then resuspended in PBS by sonicating with 20 pulses at 0.5 s/pulse using a hand-held QSonica probe and then centrifuged again at 45,000 g for 30 min at 4 °C. The pellet was resuspended in one-fifth the previous PBS volume and sonicated with 20–60 pulses and centrifuged 10,000 g for 30 min at 4 °C. The final supernatant contained enriched AD-tau at 5–20% purity. Total tau concentration was determined using a Tau5 ELISA assay as previously described [17].

### Tau expression, purification, and seeded fibrillization

Full length tau protein, T40 (2N4R) isoform, and fragments were expressed in BL21(DE3)RIL *E. coli* and purified by cation exchange chromatography as previously described [47]. AD-tau seeded recombinant tau PFFs (i.e., AD-P1 PFFs) were generated by incubation of 36 μM T40 tau (2N4R) with 4 μM AD-tau seeds comprising 10% of the fibrillization reaction with 2 mM DTT in PBS pH 7.0, with shaking at 1000 RPM for 3 days at 37 °C as previously described [21]. The reaction mixture was centrifuged at 45,000 g for 30 min and fibrillized tau was collected in the pellet fraction, which was resuspended in the original reaction volume mixture.

### Antibody generation

DMR7 and SKT82 hybridoma clones were generated as previously described for other tau mAbs [48]. Briefly, mice were injected subcutaneously with AD-tau in Freund’s complete adjuvant, and spleens were dissociated and fused with SP2 myeloma cells with PEG treatment. Hybridoma clones were diluted with limiting dilutions and screened for tau antibody binding to T40 tau, AD-P1 PFFs, and AD-tau. Clones with selective binding to AD-P1 PFFs and AD-tau were prioritized and sub-cloned two times to ensure monoclonal populations. Control murine IgG1 antibody, CHL34, is directed against the herpes simplex virus 2 envelope glycoprotein L [49] and murine IgG2b antibody, 52S, is directed against herpes simplex virus 1 glycoproteins H/L [50]. Antibodies were purified from hybridoma cell culture media using HiTrap MabSelect SuRe columns (GE Healthcare). All procedures were approved by the University of Pennsylvania Institutional Animal Care and Use Committee (IACUC) and performed according to the NIH Guide for the Care and Use of Experimental Animals.

### Sandwich ELISA

Rabbit polyclonal anti-tau antibody K9JA (Dako) was coated onto 384-well MaxiSorp plates (Fisher Scientific) at 100 ng/well in 0.1 M sodium carbonate buffered to pH 9.6 at 4 °C overnight. Blocking was performed with BlockAce solution (Abd Serotec) at 4 °C overnight. Fibrillized tau antigens AD-P1 PFFs and AD-tau were sonicated, and then all tau antigens including T40 monomer were diluted to 0.2–0.8 μg/mL in 0.2% bovine serum albumin (BSA) in PBS and applied to K9JA-coated plates. Antigens were captured by total tau antibody K9JA at 4 °C overnight. Plates were washed with PBS containing 0.015% Tween-20 (PBST) and the novel mouse tau antibodies or Tau5 total tau control antibody were added to plates for 2 h at 22 °C. Plates were washed with PBST and HRP-conjugated anti-mouse (Jackson Immunoresearch) secondary antibodies were applied for 2 h at 22 °C. Plates were washed and tetramethylbenzidine peroxidase substrate (KPL laboratories) was added to wells to provide a colorimetric readout, quenched with 10% phosphoric acid and then absorbance measured at 450 nm (Molecular Devices SpectraMax).

### Tau denaturation and immunoblots

T40 tau monomer or AD-tau were chemically and thermally denatured by 1:10 dilution in 8 M guanidine hydrochloride and heating at 100 °C for 15 min, and non-denatured controls were diluted in Tris-buffered saline (TBS) at RT. Denatured or non-denatured tau was then diluted 1:50 in TBS and applied to 0.2 μm nitrocellulose membrane using a vacuum apparatus. Each dot of recombinant tau monomer was loaded with 0.25 μg tau and AD-tau was titrated to determine equivalent loading based on detection of total tau with K9JA antibody. For western blots, tau isoforms and fragments were diluted in SDS sample buffer, heated 10 min at 100 °C, run on 12.5% SDS-PAGE gels and transferred to 0.2 μm nitrocellulose membrane. Immunoblots and dot blots were probed with either total tau control antibody K9JA diluted to 2 μg/mL or conformation-selective mAbs diluted in 5% non-fat milk at 20 μg/mL at 4 °C. Infrared dye labeled secondary antibodies (LiCor) were used to detect primary antibody binding, with analysis on a LiCor scanner (LiCor).

### Immunohistochemistry (IHC)

All human brain tissue samples used in this study were obtained at autopsy, fixed in ethanol or paraformaldehyde, paraffin-embedded, and cut into 6 μm thick sections and characterized as described [51, 52]. For IHC staining, sections were deparaffinized in xylene and rehydrated in ethanol (100–70%) as previously reported [48]. Tau antibodies MC1 and PHF-1 (gift of Peter Davies) were diluted 1:1000 and 1:5000, respectively, while DMR7 and SKT82 were diluted to 2 μg/mL in 2% FBS in 50 mM Tris pH 7.2 and applied overnight to rehydrated tissue sections without antigen retrieval in a humidified chamber at 4 °C. Antibody binding was detected by Vectastain Elite ABC Kit (Vector) followed by DAB peroxidase substrate (Vector) and counterstaining with Harris hematoxylin (ThermoFisher).

### Seeded aggregation of tau in primary neurons

Primary cortical neurons from E16–19 CD-1 WT mice were cultured in 96-well plates at a density of 17,500 cells/well. After 7 days in vitro (DIV 7), the cells were treated in triplicates with either SKT82, DMR7, or IgG with at concentrations of 3.3–26.7 nM and subsequently treated with 0.125 μg of AD-tau per well. After 7 days of AD-tau and antibody treatment, neurons were maintained in growth factor-rich conditioned media until DIV 21. Cells were washed with PBS and soluble tau was extracted with 0.5% HDTA for 15 min at RT, followed by fixation with 4% PFA and 4% sucrose for 15 min at RT. Immunocytochemistry was performed with the mouse tau-specific R2295M (CNDR) antibody, fluorescently labeled with anti-rabbit secondary antibody, and DAPI to stain nuclei. Imaging was performed on an InCell Analyzer 2200 microscope. The DAPI-positive cell count, and area and density of mouse tau pathology, were analyzed using the InCell Developer Toolbox software. The final quantification was based on density × area of pathological insoluble mouse tau normalized to cell number by DAPI-positive nuclei. Colocalization of mouse tau pathology and MAP2 was assessed by immunofluorescent staining of pathology with the mouse tau specific antibody T49 (CNDR) co-stained with MAP2 (Millipore) antibody. Confocal microscopy was performed on a Leica DMI 6000 microscope.

### Immunoprecipitation

A mixture of Dynabeads conjugated with protein A and protein G were combined with 10 μg of purified DMR7, SKT82, or IgG control antibody and AD-tau containing 2 μg of tau protein and rotated for 3 h at RT. A magnetic stand was used to sequester Dynabeads and supernatant was transferred to another tube as the unbound fraction. Beads were washed three times with PBS and bound proteins eluted with SDS sample buffer and heating at 100 °C for 10 min. Equal proportions of unbound reaction and eluted proteins were run on 10% SDS-PAGE gels, transferred to nitrocellulose membranes and immunoblotted with the rabbit polyclonal total tau antibody 17,025 (CNDR in-house).

### pH-sensitive tau labeling and cellular uptake experiments

Purified recombinant T40 tau (2N4R) was labeled with the pH-sensitive fluorescent tag, pHRodo red, by incubating 18 μM T40 tau with 10-fold molar excess of pHRodo red succinimidyl ester (ThermoFisher) for 1 h at RT. Excess dye was removed by dialysis and fluorescently-labeled tau, termed pHR-T40, was used in an in vitro seeded fibrillization reaction consisting of 10% pHR-T40, 80% unlabeled T40, and 10% sonicated AD-tau seeds. The reaction was shaken at 1000 RPM for 4 days at 37 °C and fibrils collected by centrifugation at 100,000 g for 30 min. The pellet fraction containing pHR-T40 AD-seeded preformed fibrils (pHR-T40 AD-P1 PFFs), was resuspended in PBS and sonicated. Tau mAbs were preincubated with pHR-T40 AD-P1 PFFs for 30 min at 37 °C in conditioned neuronal media and added to E16-E18 cultured cortical neurons at DIV7–10. After 24 h treatment with pHR-T40 AD-P1 PFFs, live cells were treated with NucBlue to stain nuclei and internalized pHR-T40 AD-P1 PFFs were imaged on a Leica DMI6000 microscope or imaged and evaluated with an InCell2200.

### Slice culture model of tau pathology

Organotypic hippocampal slice cultures were prepared from postnatal (P) 10–12 C56BL/6 J as previously described [53, 54] with minor modifications. Briefly, pups were sacrificed and dissected hippocampi were kept in ice-cold Krebs buffer 1.2 mM NaH2PO4, 126 mM NaCl, 2.5 mM KCl, 1.2 mM MgCl2, 2.5 mM CaCl2, 25 mM NaHCO3, 10 mM D-glucose, 10 mM HEPES, 1% (v/v) penicillin/streptomycin pH 7.4. Next, 350 μm thick coronal slices were cut using a McIlwain Tissue Chopper (Stoelting, VWR) and 5–6 consecutive slices were positioned on Millicell culture inserts (MilliporeSigma) for culture in 6-well plates (ThermoFisher Scientific). On DIV 7, AD-tau and tau mAbs were added as in primary neuron cultures described above. Two days later, the medium was changed completely with pre-warmed fresh culture medium. On DIV 35 slices were fixed for 20 min in 4% paraformaldehyde (PFA) in dPBS. Slices were washed twice in dPBS, blocked for 1 h in blocking solution (3% fetal bovine serum, 0.5% Triton X-100 in dPBS) then incubated in 200 μL primary antibody AT8 (ThermoFisher Scientific) diluted 1:1000 in blocking solution overnight at 4 °C. Slices were washed 3 times in dPBS before being incubated for 2 h at room temperature in the dark with Alexa 488-conjugated secondary antibodies (Life Technologies). Slices were washed 3 times in dPBS and counterstained with DAPI. Images were captured on an LSM 710 inverted confocal microscope (Carl Zeiss). Background subtraction, z-stack projection, and fluorescence intensity analysis were acquired with ImageJ software (National Institutes of Health, USA).

### Direct ELISA quantification of tau mAbs in CSF and plasma

AD-tau was coated onto 384-well MaxiSorp plates (Fisher Scientific) at 30 ng/well in 0.1 M sodium carbonate buffered to pH 9.6 at 4 °C overnight. Plates were blocked with BlockAce solution (Abd Serotec) at 4 °C overnight. CSF was collected from the cisterna magna of anesthetized mice following intraperitoneal injection of ketamine/xylazine. CSF samples were flash frozen on dry ice and kept at − 80 °C until analysis. Plasma was obtained from cardiac puncture blood treated with EDTA and centrifuged 2000 g 15 min 4 °C. CSF and plasma were incubated in AD-tau coated plates 2 h at RT and detected with HRP-conjugated anti-mouse secondary antibodies (Jackson Immunoresearch). Tau mAbs levels in CSF and plasma were determined by comparison to standard curves of purified DMR7 and SKT82.

### In vivo assessment of tau transmission

Four month old female 5xFAD mice were stereotaxically-injected with 2 μg AD-tau or 4 μL PBS into the hippocampus (Bregma: − 2.5 mm; lateral: + 2 mm; depth: − 2.4 mm from the skull) as previously described [55]. Mice were injected (intraperitoneal or i.p.) with 60 mg/kg of tau mAbs or IgG control mAb 4 days prior to AD-tau injection, on the day of AD-tau injection, and weekly thereafter for 3 months (PBS, *n* = 12 IHC, *n* = 2 biochemistry; IgG1, *n* = 9 IHC, *n* = 3 biochemistry; DMR7, *n* = 10 IHC, n = 3 biochemistry; IgG2b, *n* = 13 IHC, *n* = 3 biochemistry; SKT82, *n* = 11 IHC, *n* = 3 biochemistry). Upon completion of the dosing period, mice were transcardially perfused with 30 mL phosphate buffered saline (PBS) at 120 mL/h and brains were fixed in 4% paraformaldehyde overnight. These procedures were approved by the University of Pennsylvania Institutional Animal Care and Use Committee (IACUC) and performed according to the NIH Guide for the Care and Use of Experimental Animals. Fixed brains were embedded into paraffin blocks and then sectioned into 6 μm sections. To minimize experimental variation of AT8 staining, multiple mice from varying treatment groups were grouped together on individual slides and thus each slide contained 6 mouse brains at equivalent Bregma levels. IHC staining with AT8 was performed on the entire study cohort of mice simultaneously to avoid experimental variation in antibody dilution or DAB development time. Every 20th section through the hippocampus was subjected to IHC staining with AT8 mAb (ThermoFisher) diluted at 0.04 μg/mL or alternatively with 50 μM X-34, an amyloid binding dye (Sigma Aldrich). Slides were scanned with a 20x objective using a Lamina slide scanner (PerkinElmer). AT8 IHC staining was quantified in a blinded manner with randomized treatment groups represented on each slide. The hippocampus and dentate gyrus of each mouse were annotated and AT8 positive area was quantified by applying the same AT8 positive threshold across all animals using Halo software (Indica Labs) .

### Sequential extraction of soluble and insoluble tau from mouse tissue

Brains were separated into ipsilateral and contralateral hemispheres and hippocampus was dissected and flash frozen on dry ice and kept at − 80 °C. Tissue was homogenized by sonication in 9 volumes of HS-RAB buffer (100 mM MES, 1 mM EDTA, 0.5 mM MgSO_4_, 1 mM DTT, 1 mM PMSF, 0.75 M NaCl, 20 mM NaF, pH 6.8) with protease inhibitors and phosphatase inhibitors. Homogenates were centrifuged at 45,000 g for 30 min at 4 °C and the supernatant was saved as the soluble fraction, while the pellet was resuspended in 9 volumes of HS-RAB buffer containing 1% Triton X-100. Samples were again centrifuged (45,000 g for 30 min at 4 °C) and the supernatants were discarded, while the pellet was resuspended in 9 volumes of HS-RAB buffer containing 1% sarkosyl and then rotated for 1 h at 22 °C. Samples were centrifuged at 45,000 g for 45 min at 4 °C, the supernatant discarded, and the pellet washed with 500 μL PBS. Samples were centrifuged (45,000 g 30 for min at 4 °C), the supernatant discarded, and the pellet was resuspended in PBS at a volume two-times the mass of the original tissue and sonicated to resuspend as the insoluble fraction.

### Behavioral tests

All animals were cared for in accordance with the guidelines of the National Institutes of Health and procedures were approved by the University of Pennsylvania Institutional Animal Care and Use Committees. Open Field procedures were horizontal and vertical activity recorded with IR beam breaks during a 10 min trial. Data were acquired with a Photobeam Activity System (San Diego Instruments). The percent spontaneous alternation in the Y-maze was calculated as 100 x [number of alternations / (total arm entries− 2)] where arm entry was defined as all four paws placed inside an arm. For contextual fear conditioning, a mouse was placed in a conditioning chamber (Med Associate) within a sound-attenuating cabinet, and a 2-s, 1.75 mA foot shock was delivered at 148–150 s of a 180-s acquisition trial. Twenty-four hours after acquisition, mice were returned to the conditioning chamber for a 5 min recall trial to assess long-term memory. Fourteen days after acquisition, remote memory was also assessed with a second recall trial. All trials were digitally recorded. Time spent motionless was automatically assessed by Freezescan software (Clever Systems).

### Statistical analysis

Statistical analyses were performed with GraphPad Prism software. Quantification of tau uptake in primary neurons and tau mAb dose response inhibition of tau pathology in the seeded aggregation model in primary neurons and slice culture were evaluated by One-way ANOVA with Dunnett’s post-hoc analysis. Quantification of AT8 positive tau pathology in vivo were analyzed using two-tailed unpaired t-test in which DMR7 or SKT82 treated mice were compared directly to the respective IgG1 or IgG2b isotype control. Contextual fear conditioning measures of % freezing consisted of repeated measurements of the same mice at different time points and were analyzed by two-tailed paired t-test to compare changes from prestimulation to 24 h recall and 24 h recall to 14 day remote recall.

## Results

### Generation of conformation-selective tau mAbs

**Fig. 1:**
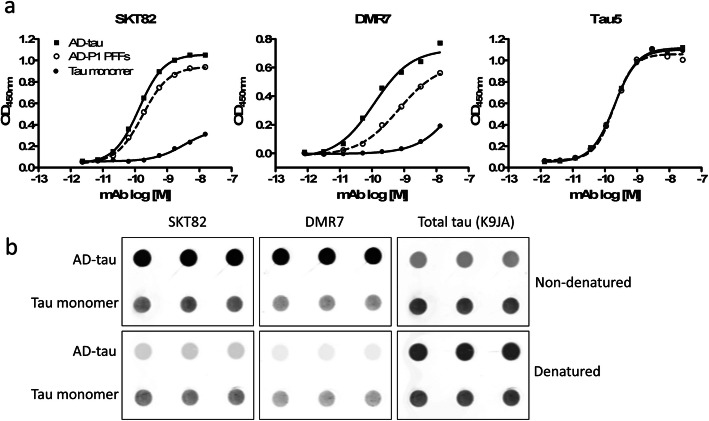
Novel tau mAbs selectively bind AD-tau compared to tau monomer. **a** Sandwich ELISA assay comprised of total tau capture antibody K9JA and three distinct tau antigens, AD-tau, AD-P1 PFFs, and tau monomer, detected by novel tau mAbs DMR7 or SKT82 and total tau antibody Tau5 as a loading control demonstrating similar levels of captured antigen for each form of tau. **b** Dot blot assay of AD-tau and tau monomer immobilized onto nitrocellulose membrane without treatment or denatured by guanidine hydrochloride and heat treatment. Total tau immunoblotted by K9JA shows similar levels of tau immobilization. DMR7 and SKT82 selectively detect AD-tau compared to monomer and binding is diminished by denaturation, demonstrating that the conformation of pathological AD-tau is responsible for enhanced binding

Towards the goal of generating conformation-selective tau mAbs, mice were inoculated with AD-tau [21, 22]. Hybridoma clones generated from immunized mice were screened by sandwich ELISA to simultaneously assess binding of antibodies to tau monomer, AD-tau seeded recombinant tau preformed fibrils (AD-P1 PFFs), and AD-tau. The pan-tau antibody Tau5 served as a loading control to ensure equivalent capture of the three tau forms by the immobilized pan-tau K9JA capture antibody. Clones that bound AD-tau and AD-P1 PFFs with greater apparent affinity than tau monomer were prioritized and subsequently sub-cloned by limiting dilutions to monoclonal populations. We identified two novel tau mAbs, DMR7 and SKT82, that demonstrated increased affinity to AD-tau and AD-P1 PFFs compared to tau monomer (Fig. 1a). Sandwich ELISA measures of DMR7 provided an EC_50_ of 0.10 + 0.01 nM for AD-tau, 0.46 + 0.32 nM for AD-P1 PFFs and 12.0 + 7.9 nM for tau monomer, whereas SKT82 EC_50_ values were 0.17 + 0.03 nM for AD-tau, 2.38 + 1.12 nM for AD-P1 PFFs, and 4.13 + 3.74 nM for tau monomer. Lastly, Tau5 is a control antibody that binds to a linear epitope of tau in the central proline-rich domain and provides EC_50_ of 0.093 + 0.043 nM for AD-tau, 0.138 + 0.125 nM for AD-P1 PFFs, and 0.103 + 0.065 nM for tau monomer. The EC_50_s of Tau5 reflect comparable binding to each form of tau and supports the observation that differences in EC_50_ observed with DMR7 and SKT82 result from selective binding to AD-tau and AD-P1 PFFs compared to tau monomer.

To assess whether conformational differences between AD-tau and tau monomer contributed to the differences observed by ELISA, AD-tau and tau monomer were chemically and thermally denatured prior to immobilization on dot blots and probing with the DMR7 and SKT82 mAbs (Fig. 1b). Consistent with the ELISA results, both DMR7 and SKT82 showed greater interaction with non-denatured AD-tau than tau monomer, with loading of total tau assessed by binding of the K9JA pan-tau antibody. Upon denaturation of tau, the signal for DMR7 and SKT82 binding to AD-tau was greatly diminished, whereas the tau monomer signal was unaffected. Denaturation did not reduce the immobilization of AD-tau or tau monomer, as evidenced by similar or even slightly greater binding of K9JA to denatured tau compared to non-denatured tau. This demonstrates that denaturation of the AD-tau conformation reduces binding of DMR7 and SKT82 and suggests that the selectivity observed by ELISA results from binding to the misfolded pathological conformation of AD-tau.

### Novel tau mAbs detect pathological tau in multiple tauopathies

**Fig. 2:**
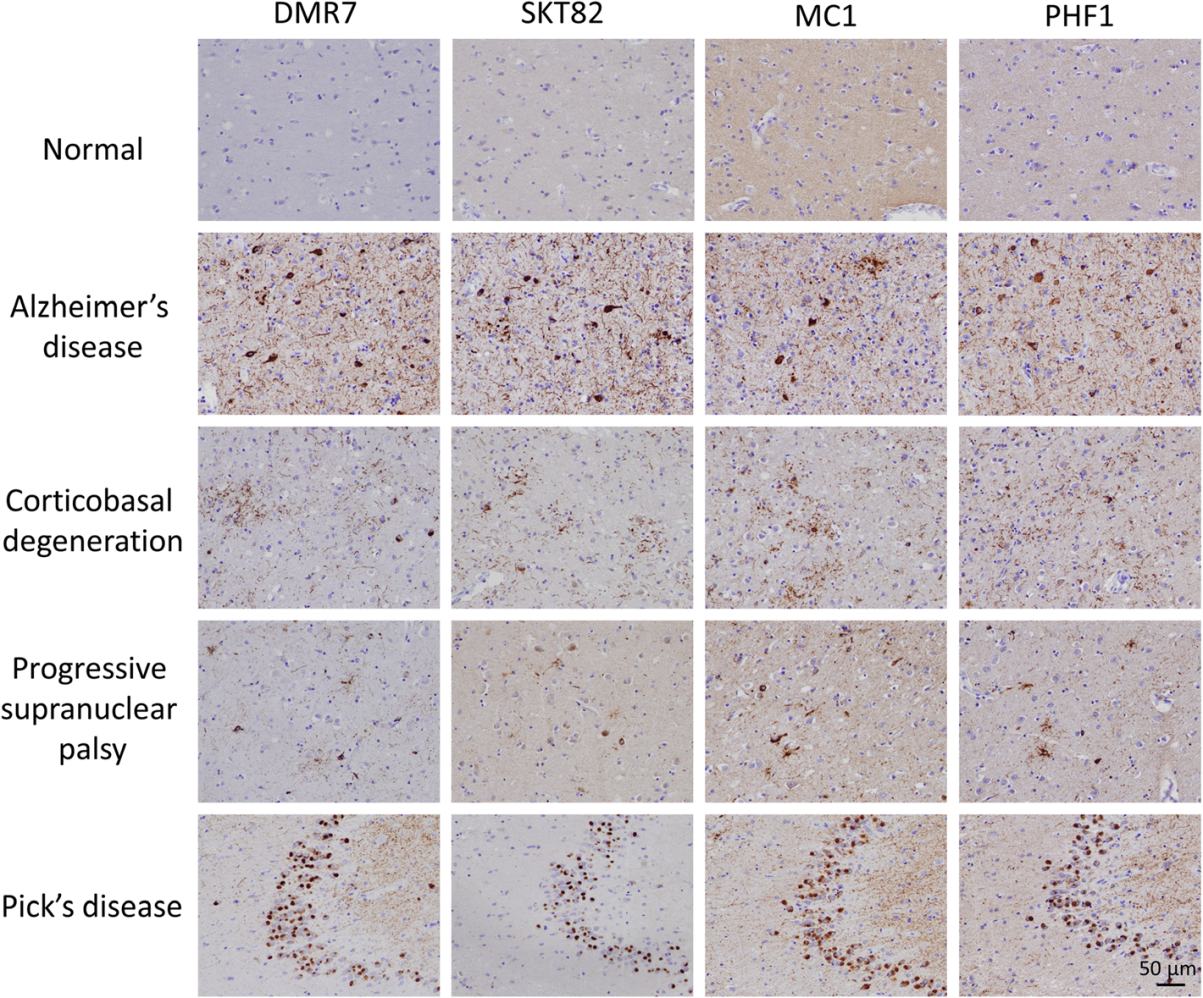
Conformation-selective tau mAbs detect multiple human tauopathies. Immunohistochemical staining with novel conformation-selective tau mAbs, DMR7 and SKT82, conformation-selective control mAb MC1, and phospho-tau mAb PHF1, demonstrates binding of DMR7 and SKT82 to pathological tau in AD frontal cortex, CBD cingulate cortex, PSP lentiform nucleus, and PiD hippocampus and dentate gyrus but not frontal cortex of cognitively normal control

To determine whether DMR7 and SKT82 detect various forms of pathological tau, we analyzed brain tissue from several human tauopathies by IHC, including AD, corticobasal degeneration (CBD), progressive supranuclear palsy (PSP), and Pick’s disease (PiD). Both mAbs detected pathological tau lesions in all of the tauopathy brain sections to a similar extent as the diagnostic standard PHF1 antibody, which binds tau phosphorylated at Ser396 and Ser404 (Fig. 2). In AD midfrontal cortex, NFT, NP, and neuropil thread (NT) staining were readily detected by DMR7 and SKT82. In CBD cingulate cortex, astrocytic plaques and neuronal cell body pathology were detected by both mAbs. In PSP lentiform nucleus, glial astrocytic plaques and oligodendroglia coiled bodies were detected by DMR7 and SKT82, in addition to neuronal inclusions. Lastly, abundant round “Pick bodies” were readily detected in the dentate gyrus of PiD brain tissue by the two mAbs. Thus, both DMR7 and SKT82 differ from previously described conformation-preferring tau antibodies that selectively detected pathological tau in AD but not in other tauopathies [48]. In fact, DMR7 and SKT82 staining of the various tauopathies resembles that observed with the well characterized conformation-selective antibody MC1 [42] (Fig. 2) which, as previously reported, detected misfolded pathological tau in all four tauopathies. As growing in vivo and structural data demonstrate that different tauopathies are comprised of distinct conformational strains [22], these data demonstrate that DMR7 and SKT82 bind to a conformational epitope that is common among the tau strains found in the tauopathies tested here. Since the tau fibril cryo-EM structures to date primarily resolve the fibrillization core domain comprised of microtubule binding repeats [4, 56–58], we speculate that the epitopes involved in our conformation-selective mAb binding are either shared among these different tau fibril structures or that these different fibril structures have some flexibility that can accommodate mAb binding despite unique fibril core domains.

### DMR7 and SKT82 bind conformational epitopes of tau

**Fig. 3:**
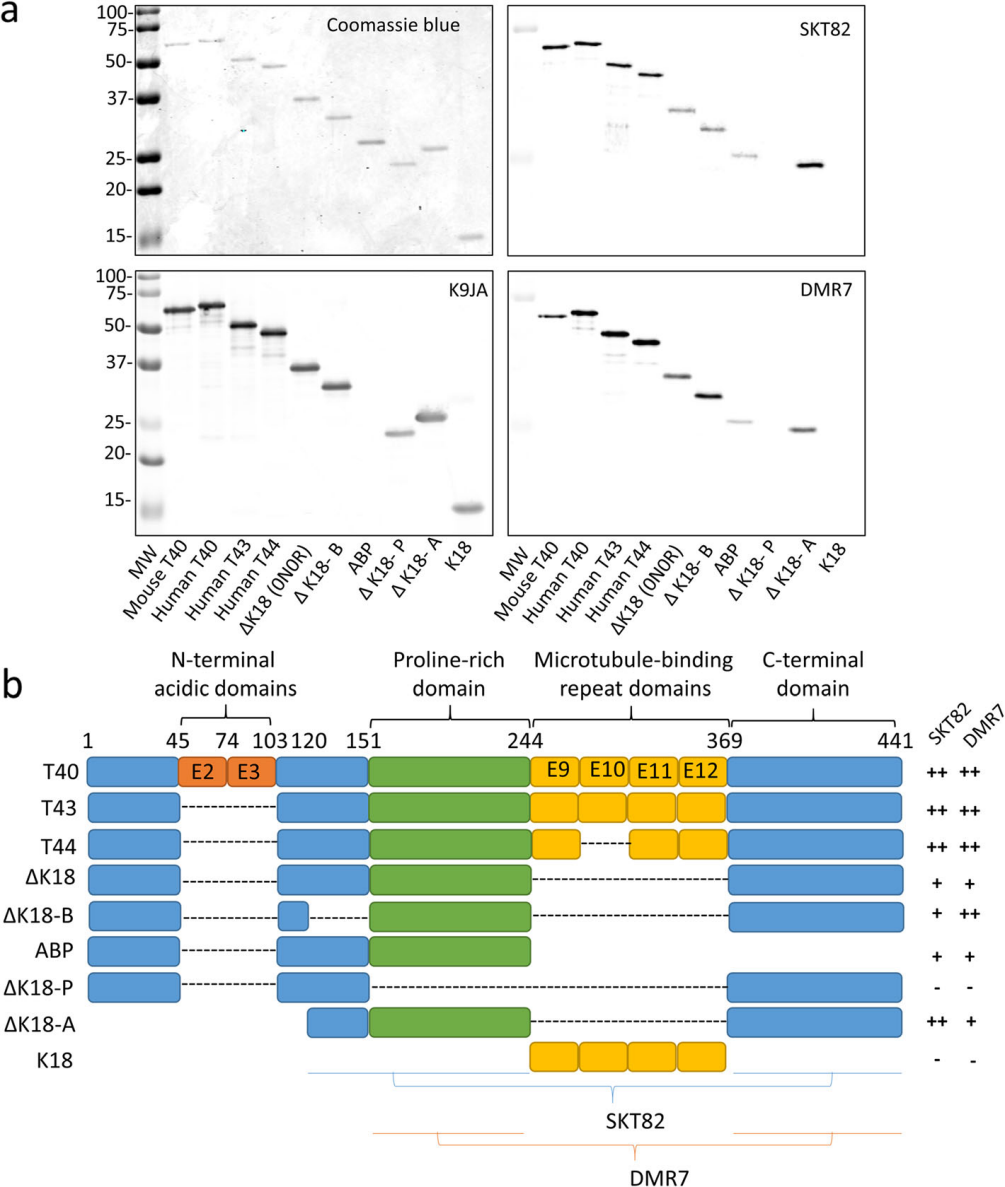
DMR7 and SKT82 bind to discontinuous epitopes of tau. **a** Western blot of tau fragments with DMR7 and SKT82 reveal distinct partial binding patterns to tau fragments. DMR7 and SKT82 detect full length tau isoforms T44, T43, and T44, but not the microtubule binding domain, K18. Loss of the C-terminus in the ABP construct and loss of the proline rich domain in the ΔK18-P construct reduce binding of DMR7 and SKT82, demonstrating a proline-rich domain and c-terminal epitope. Equal loading of tau protein fragments was determined by Coomassie blue stained gel and K9JA total tau antibody, which does not detect the ABP fragment. **b** Schematic of tau constructs and tau mAb binding

To gain insight into the nature of the conformation selectivity of DMR7 and SKT82, we sought to verify the epitope of these mAbs. Consistent with previously reported conformation-selective tau mAbs such as MC1 [42], DMR7 and SKT82 were able to detect full-length tau monomer by immunoblot even though they have a relatively low affinity for monomers by ELISA. Therefore, we examined a panel of tau fragments to identify those containing binding epitopes of DMR7 and SKT82 (Fig. 3a). Coomassie blue staining of the gels revealed comparable loading of all the tau fragments, which was further verified with the K9JA antibody with the exception of the ABP fragment, which is missing the K9JA C-terminal epitope. DMR7 detected all full-length tau isoforms including mouse tau but showed some selectivity for human T40 tau compared to mouse T40 tau, whereas SKT82 detected both full-length mouse and human tau isoforms similarly. Other than the slight differences in species selectivity, DMR7 and SKT82 have a number of similarities between their epitope profiles. Both tau mAbs failed to bind to the K18 fragment of tau, demonstrating the epitope is not in the MTBR region comprising the fibrillar core of tau PHFs (Fig. 3b). Consistent with this observation, both tau mAbs showed binding to the ΔK18 fragment which lacks the MTBRs; however, binding of both mAbs is slightly diminished compared to the binding of 4R tau isoforms. Deletion of the proline-rich domain between amino acids 151–244 (ΔK18-P) abolished binding of both mAbs, demonstrating an essential epitope within that region. The ABP fragment of tau, which contains the proline-rich region but lacks the C-terminus, also demonstrated greatly diminished binding by the mAbs, suggesting that optimal mAb binding requires a C-terminal epitope in addition to the proline-rich domain epitope. Both DMR7 and SKT82 bound well to ΔK18-A, which lacks the N-terminus and MTBRs and consists of the basic region from 120 to 151, the proline rich domain, and the C-terminus. Lastly, comparison of the SKT82 signal between ΔK18-A and ΔK18-B suggests that SKT82 may bind an epitope in the basic region from 120 to 151. Together, these findings suggest that DMR7 and SKT82 bind to conformational epitopes of tau comprised of the proline-rich domain containing amino acids 151–244 and a C-terminal epitope from amino acids 369–441. Additionally, SKT82 may have some binding contribution from the basic region containing amino acids 120–151.

### Tau mAbs inhibit seeded fibrillization of endogenous tau in primary neurons

**Fig. 4:**
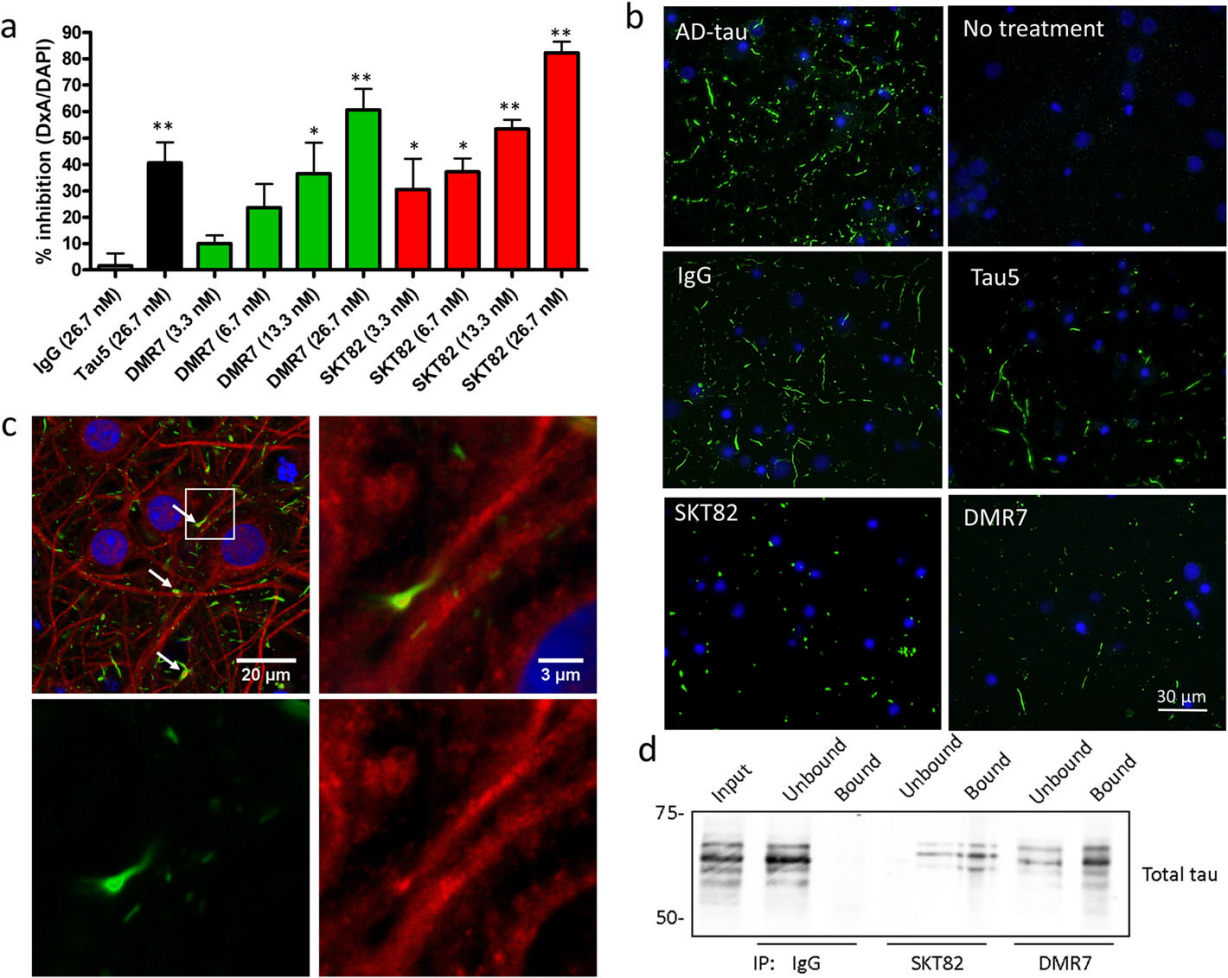
Tau mAbs inhibit AD-tau seeded aggregation of endogenous mouse tau in primary neurons. **a** Quantification of immunocytochemistry detection of AD-tau seeded insoluble mouse tau in primary neurons detected by the mouse-tau specific R2295M antibody. Statistical significance was determined relative to non-specific IgG control, using one-way ANOVA with Dunnett’s post-hoc analysis; *p < 0.05, **p < 0.01, *n* = 3–4 biological replicates each consisting of 3 technical replicate wells per plate. **b** Representative immunofluorescent staining images of insoluble mouse tau aggregates in primary neurons induced by AD-tau seeding detected by the mouse tau specific rabbit polyclonal antibody R2295M (green) and DAPI (blue). Tau5, DMR7, and SKT82 show inhibition of seeded tau pathology. **c** Confocal microscopy images of AD-tau induced aggregates of mouse tau in primary neurons detected by the mouse tau specific antibody T49 (green) and dendritic processes stained with MAP2 (red) with nuclei stained with DAPI (blue). Areas of MAP2 and mouse tau aggregate colocalization are indicated by white arrows. **d** Immunoprecipitation of tau from AD-tau extracts by IgG, SKT82, or DMR7. Bound and unbound fractions evaluated by western blot with total tau antibody (17025)

To determine whether DMR7 and SKT82 binding to AD-tau would inhibit the seeding of tau aggregates in primary neurons in a previously described assay [21], WT mouse cortical neurons were treated with AD-tau to template fibrillization of endogenous cellular mouse tau into insoluble aggregates. Both DMR7 and SKT82 significantly reduced the seeding of pathological tau aggregates in a dose-dependent manner (Fig. 4a and b), with mouse tau pathology inhibited by 60.5 + 13.8% with DMR7 treatment and 82.2 + 8.3% by SKT82 addition. Both conformation-selective tau mAbs provided greater inhibition of AD-tau seeding in primary neurons than the pan-tau control antibody, Tau5. As expected, non-specific mouse IgG control antibody did not inhibit cellular tau aggregates induced by AD-tau. To demonstrate that the insoluble mouse tau aggregates induced by AD-tau are intracellular, we examined the colocalization of the mouse tau specific antibody,T49, with the neuronal dendritic marker MAP2. Although there is not complete colocalization of these markers, consistent with previous findings [21], confocal microscopy demonstrates that numerous mouse tau aggregates colocalize with MAP2 and in some cases are adjacent, suggesting that AD-tau seeded mouse tau aggregates are intraneuronal (Fig. 4c). Furthermore, both DMR7 and SKT82 immunoprecipitated tau from the complex protein mixture present in the AD-tau extracts (Fig. 4d), supporting the notion that conformation-selective tau mAbs inhibit seeded aggregation in tau primary neurons through direct sequestration of tau seeds.

### DMR7 and SKT82 block uptake of labeled AD-P1 PFFs into neurons

**Fig. 5:**
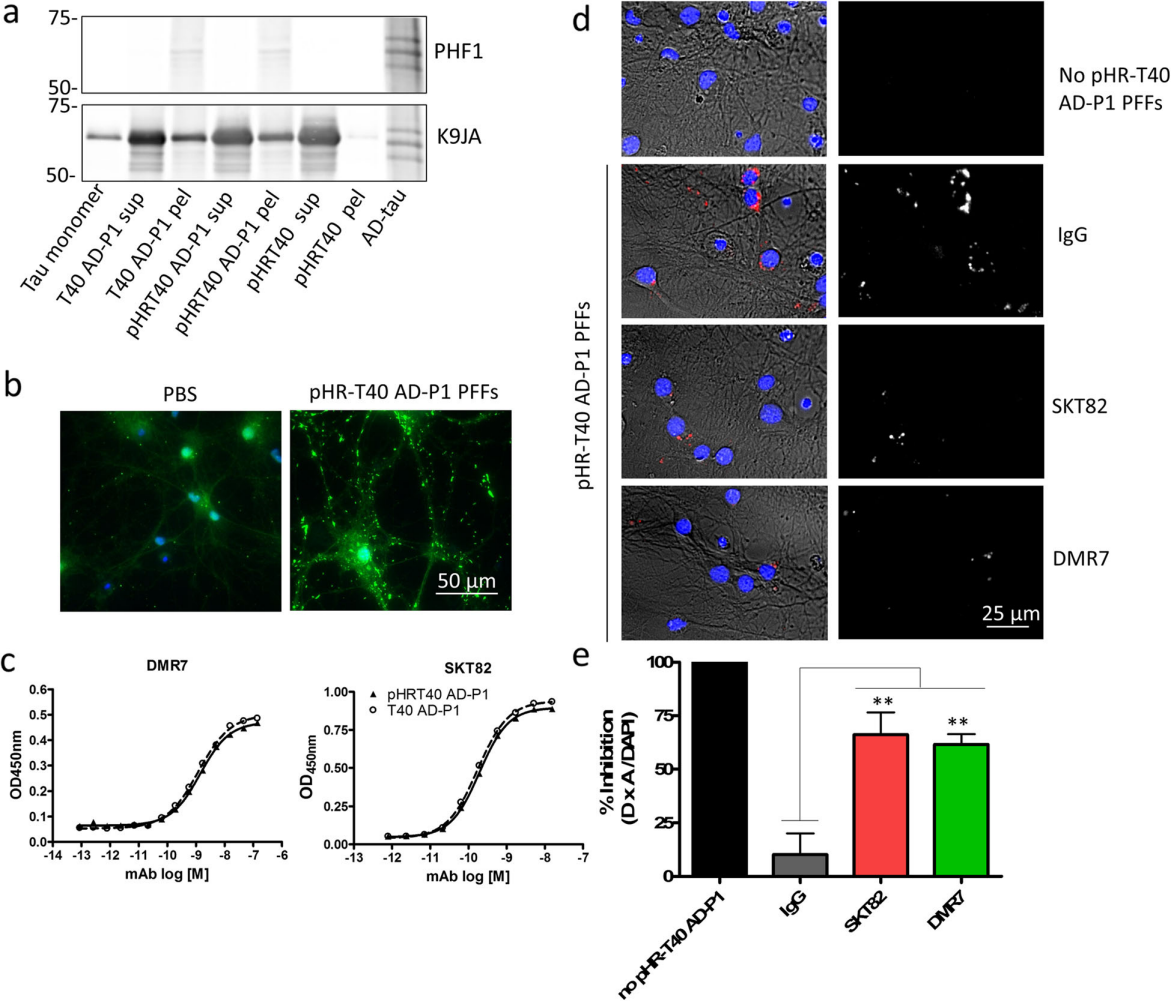
Tau mAbs inhibit uptake of tau seeds into primary neurons. **a** Western blot characterization of AD-tau seeded fibrillization reaction sedimentation following centrifugation of unlabeled tau monomer (T40 AD-P1), pHR-T40 AD-P1 fibrillization reaction, and pHR-T40 monomer lacking AD-tau seeds. T40 tau monomer and AD-tau are included as controls. **b** Immunocytochemistry staining of mouse-tau specific R2295M antibody (green) demonstrating that pHR-T40 AD-P1 PFFs are capable of seeding pathological tau aggregates in WT primary mouse neurons. **c** Sandwich ELISA assay of pHR-T40 AD-P1 and T40 AD-P1 PFFs captured by total tau antibody K9JA and detected with varying concentrations of SKT82 or DMR7. **d** Immunofluorescence of internalized tau fibrils labeled with pH-sensitive pHRodo-red dye that fluoresces in acidic late endo/lysosomal compartments. Left panels: overlay of brightfield, pHR-T40 AD-P1 PFFs red channel, and DAPI nuclei blue channel. Right panelsl: pHR-T40 AD-P1 PFFs red channel converted to white for visualization. **e** Quantification of fluorescent internalized pHRodo-red-labeled tau fibrils. Non-specific mouse IgG control did not inhibit uptake of fibrils into neurons, whereas SKT82 and DMR7 significantly inhibited the uptake of fibrils into neurons. One-way ANOVA with Dunnett’s post-hoc analysis **p < 0.01 compared to IgG control *n* = 4 biological replicates

To elucidate the mechanism by which the conformation-selective tau mAbs inhibit seeding of cellular tau aggregates, we covalently-modified recombinant tau protein with the pH-sensitive pHRodo-red fluorophore, which fluoresces in acidic conditions including late endosomal and lysosomal compartments. Fluorophore-labeled T40 tau (pHR-T40) was fibrillized in the presence of 10% AD-tau seeds to template propagation of the AD fibril conformation (pHR-T40 AD-P1 PFFs). The pHRodo red tag did not change the seeded aggregation of pHR-T40 monomer compared to unlabeled T40 monomer, nor did the fluorescent tag induce fibrillization of pHR-T40 in the absence of AD-tau seeds (Fig. 5a). Furthermore, AD-tau seeded pHR-T40 AD-P1 PFFs are capable of seeding pathological tau aggregates in WT primary neurons (Fig. 5b). To test whether DMR7 or SKT82 binding to AD-tau seeded fibrils was influenced by the addition of the pHRodo red tag, the pelleted fractions of AD-P1 fibrillization reactions were characterized by sandwich ELISA (Fig. 5c). The binding of DMR7 and SKT82 was not influenced by the presence of the pHRodo red tag demonstrated by equivalent binding curves for each respective mAb with pHR-T40 AD-P1 or unlabeled tau T40 AD-P1. To test whether the conformation-selective mAbs inhibit the seeded aggregation of AD-tau via extracellular or intracellular binding, the cellular uptake of pHR-T40 AD-P1 PFFs was monitored by pHRodo red fluorescence in primary neurons (Fig. 5d). Both SKT82 and DMR7 significantly inhibited the uptake of pHR-T40 AD-P1 PFFs into primary neurons, with the resulting fluorescence signal decreased by 66.1 + 20.8% and 61.5 + 9.7%, respectively (Fig. 5e). This finding supports a mechanism of SKT82 and DMR7 binding to extracellular pathological tau seeds and inhibiting uptake into recipient neurons.

### SKT82 inhibits tau pathology in slice culture

**Fig. 6:**
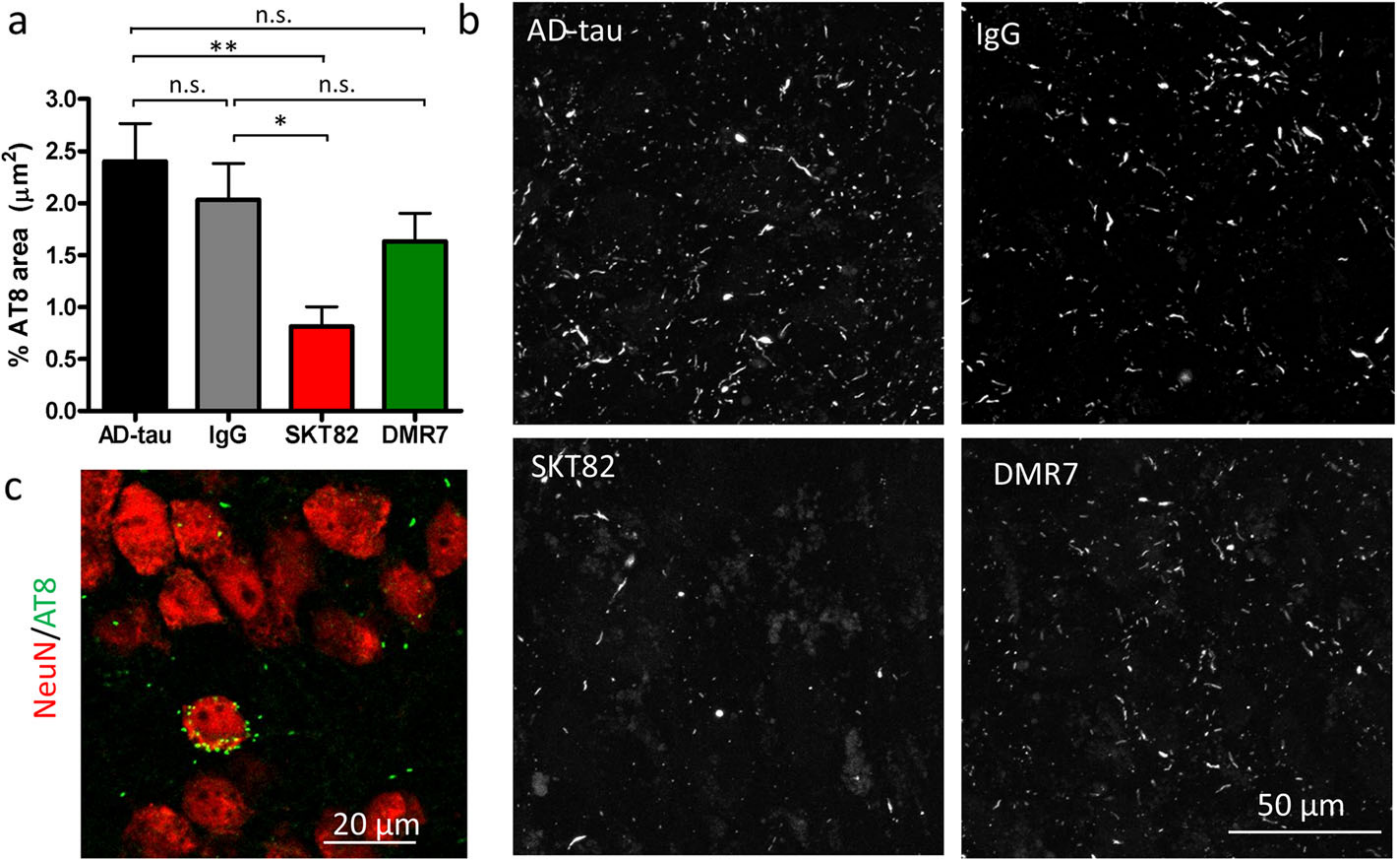
Tau mAbs inhibit seeded aggregation of tau pathology in slice cultures. **a** Quantification of immunofluorescent staining of hyperphosphorylated tau with AT8 antibody in hippocampal slice cultures treated with AD-tau and IgG control, SKT82, or DMR7. One-way ANOVA with Dunnett’s post-hoc test **p < 0.01, *p < 0.05, *n* = 6 comprised of three fields of view per slice for two independent hippocampal slices. **b** Representative images of AT8 immunofluorescent staining of tau pathology induced in the CA3 of hippocampal slices by treatment with AD-tau and inhibition by tau mAbs. **c** Confocal microscopy of the nuclear neuronal marker NeuN (red) with AD-tau induced tau aggregates stained with the phospho-tau antibody AT8 (green)

To expand on the primary neuron studies of AD-tau induced pathology, hippocampal slice cultures from WT mice were co-treated with the tau mAbs and AD-tau. Similar to our observations in primary neurons, AD-tau treatment of hippocampal slice cultures results in recruitment of endogenous mouse tau into insoluble tau aggregates that are detected with the phospho-tau antibody, AT8, particularly in the CA3 region of the hippocampus. The seeding of AT8-positive tau inclusions by AD-tau is significantly inhibited by SKT82 and there is a trend towards reduction of pathology by DMR7 that did not achieve statistical significance (Fig. 6a and b). To examine the cell-type specificity and sub-cellular localization of AT8 positive tau inclusions, hippocampal slices were co-stained with the nuclear neuronal marker NeuN and imaged with confocal microscopy. We observed that AT8 positive inclusions were present adjacent to NeuN staining, suggesting that they are both intracellular and within neurons (Fig. 6c). These studies further confirm that the conformation-selective tau mAbs can inhibit AD-tau seeding of mouse tau pathology in neurons, here utilizing a more complex culture system.

### DMR7 and SKT82 inhibit pathological tau transmission in vivo

Given the selective binding of DMR7 and SKT82 to pathological tau, as well as inhibition of AD-tau seeded aggregation in primary neurons and hippocampal slice cultures, we evaluated whether these tau mAbs inhibit the transmission of tau pathology in vivo. To confirm CNS exposure of tau mAbs, we assessed the levels of SKT82 and DMR7 in the CSF and plasma of 4-month old 5xFAD mice 4- and 7-days post-i.p. administration of antibody based on the well-established murine IgG half-life of 5 days [59]. We detected nanomolar (nM) levels of antibody in CSF and μM levels in plasma, providing a CSF/Plasma ratio from 0.12–0.31% (Table 1) that is consistent with previously published ratios of IgG molecules [46].

**Table 1:** Pharmacokinetics of tau mAb blood-brain barrier penetrance. ELISA assays of DMR7 and SKT82 levels in CSF and plasma of mice 4- and 7-days post-IP injection quantified in comparison to standard curves of purified DMR7 and SKT82

	Dose (mg/kg)	Days post injection	Plasma (μM)	Plasma %CV	CSF (nM)	CSF %CV	CSF/ plasma ratio (%)
DMR7 *n* = 3	60	4	1.29 + 0.22	17	3.78 + 0.95	25	0.31 + 0.12
DMR7 *n* = 3	60	7	1.24 + 0.45	36	2.9 + 1.9	66	0.22 + 0.08
SKT82 *n* = 2	60	4	0.91 + 0.24	26	1.20 + 0.06	5	0.14 + 0.03
SKT82 *n* = 3	60	7	0.65 + 0.19	30	0.70 + 0.16	28	0.12 + 0.06

**Fig. 7:**
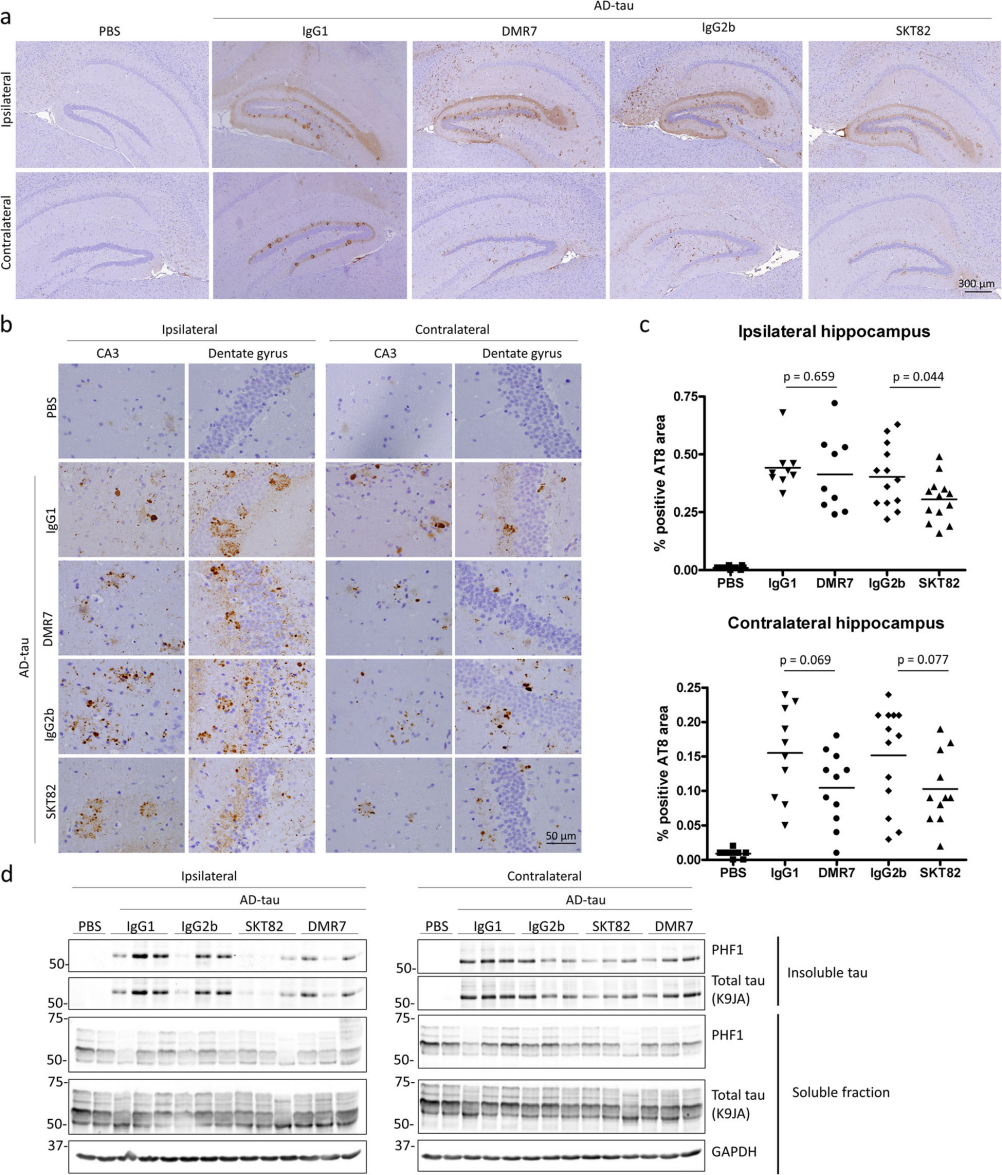
SKT82 and DMR7 inhibit tau pathology in vivo. **a** IHC staining of hyperphosphorylated tau with the AT8 antibody reveals abundant tau pathology in AD-tau injected 5xFAD mice 3 months post-injection compared to PBS-injected mice at low magnification; scale bar = 300 μm. **b** High magnification of hyperphosphorylated, AT8-positive tau pathology in the ipsilateral and contralateral dentate gyrus and CA3 region of the hippocampus demonstrating neuritic plaque tau pathology; scale bar = 50 μm. **c** Quantification of AT8-positive area reveals treatment with SKT82 significantly reduced the amount of AT8-positive tau pathology on the ipsilateral side, and DMR7 and SKT82 show a strong reduction of tau pathology on the contralateral side approaching statistical signfigance. Each mAb treatment group was individually compared to the corresponding IgG isotype control group by unpaired t-test *n* = 9–13 mice/group, each point represents mean % positive AT8 area for 3–5 sections per mouse. **d** Sequential extraction of soluble and sarkosyl-insoluble tau fractions from both ipsilateral and contralateral mouse hippocampi were evaluated for levels of hyperphosphorylated tau (PHF1) and total tau by immunoblot

Intracerebral injection of AD-tau into the 5xFAD mouse model of Aβ plaque formation induces NP tau pathology in dystrophic neurites surrounding Aβ plaques, recapitulating the hallmark plaque and tau pathology observed in AD brain [60]. In addition, seeded aggregation of tau in 5xFAD mice does not rely on transgenic overexpression of mutant human tau to develop tau pathology, as AD-tau induces fibrillization of endogenous mouse tau that accumulates in plaque-associated neuronal processes [60]. We employed a prevention model of antibody treatment in which 4-month old 5xFAD female mice received i.p. injections of 60 mg/kg of SKT82, DMR7, or isotype-matched IgG controls four days prior to unilateral injection of AD-tau into the hippocampus. Mice subsequently received the same mAb doses on the day of AD-tau injection and weekly thereafter for 3 months. Control IgG antibodies demonstrated no appreciable binding to tau by sandwich ELISA (Sup. Figure 1). We assessed the efficacy of tau mAbs to inhibit the seeded aggregation of tau pathology in vivo by measuring AT8 positive tau pathology induced by AD-tau in 5xFAD mice by IHC. We previously demonstrated that AD-tau seeds injected into mouse hippocampus are degraded below the detection threshold of AT8 IHC staining by 7-days post injection [21], therefore the pathology detected by AT8 IHC 3-months post injection represents mouse tau that has been recruited into pathological filaments. We observed a robust induction of neuritic plaque-like tau pathology in AD-tau injected 5xFAD mice whereas, PBS injected controls demonstrate very low or undetectable levels of AT8 positive tau pathology (Fig. 7a-b). We observed a strong trend toward reduced tau pathology in the contralateral hippocampus of DMR7- and SKT82-treated mice compared to their respective IgG1 and IgG2b controls, although this did not quite reach statistical significance using two-tailed unpaired Student’s t-tests (*p* = 0.069 and 0.077, respectively). There was a significant decrease in ipsilateral tau pathology in mice that received SKT82 compared to those dosed with IgG2b control mAb (*p* = 0.044), although a reduction of AT8 tau pathology was not observed in this region upon DMR7 treatment (Fig. 7c), consistent with SKT82 exhibiting greater efficacy in the slice culture model. To demonstrate that tau pathology detected by IHC is comprised of insoluble pathological tau, sequential biochemical extractions of ipsilateral and contralateral hippocampi were performed to examine insoluble and soluble tau levels by immunoblot. Probing with the PHF1 antibody, that recognizes pathological tau phosphorylated at Ser396/Ser404, we verified that AD-tau induced insoluble tau aggregates in both the ipsilateral and contralateral hippocampi compared to PBS injected controls (Fig. 7d). Although there was considerable variability between animals, SKT82 and DMR7 treatment showed a trend towards lower insoluble phosphorylated tau in the ipsilateral and contralateral sides of AD-tau injected 5xFAD mice. Taken together, these results demonstrate that SKT82 and DMR7 are able to penetrate the blood-brain barrier, thereby inhibiting the development of tau pathology in AD-tau seeded 5xFAD mice exhibiting neuropathological hallmarks of AD.

To assess whether the reduction in tau pathology was related to changes in Aβ plaque load we evaluated the plaque burden in mice using the amyloid-plaque binding dye X-34 and did not detect statistically significant difference in Aβ plaque number or area between groups (Sup. Figure 2). We also investigated whether AD-tau pathology or tau mAb treatment resulted in a neuroinflammatory response by IHC staining for microglia and astrocytes. Although Iba1-positive microglia clearly surrounded Aβ-plaques in the 5xFAD mouse model injected with PBS or AD-tau, we did not detect any overt changes in the density or morphology of either microglia or astrocytes (Sup. Figures 3 and 4).

### AD-tau pathology in 5xFAD mice did not influence behavior of 7-month old mice

**Fig. 8:**
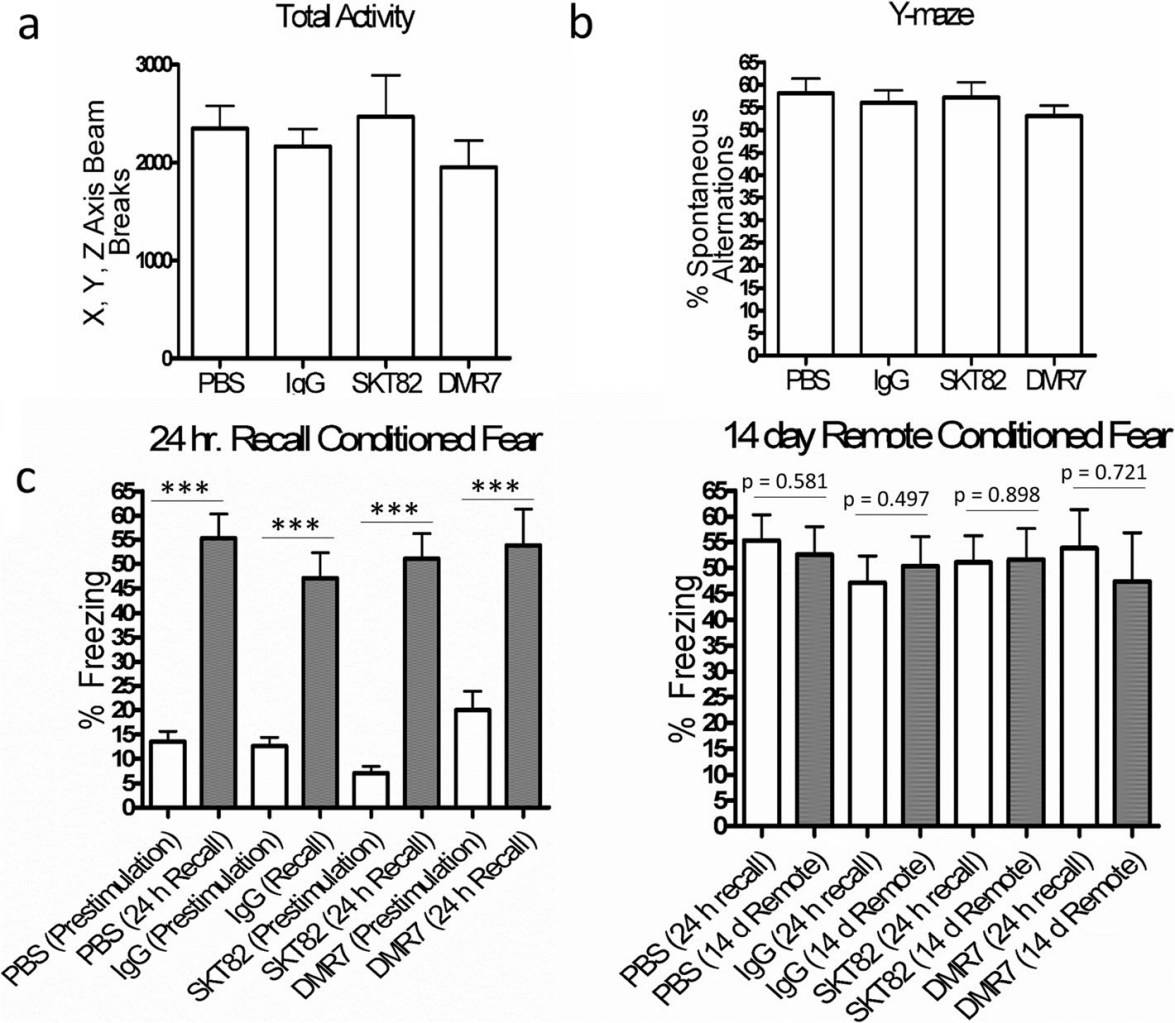
Behavioral characterization of 7-month old 5xFAD mice 3 month post-AD-tau injection. **a** Open field measurements of total activity and **b** Y-maze spontaneous alternations showed no difference between PBS controls and AD-tau-injected mice with IgG control or tau mAb treatment. Analyzed by One-way ANOVA. **c** Contextual fear conditioning showed that 5xFAD mice learned well and demonstrate a conditioned freezing response upon training but no differences at 14-day remote recall. ***p < 0.001 analyzed by two-tailed paired t-test

To assess the behavioral phenotypes of 7-month old 5xFAD mice treated for 11–13 weeks with SKT82 and DMR7 after AD-tau injection, we performed open-field, Y-maze, and contextual fear conditioning tests. SKT82 and DMR7 were generally well tolerated and did not influence total motor activity of mice in the open field test (Fig. 8a). Similarly, no changes were observed in Y-maze spontaneous alternations between PBS-injected mice without tau pathology and AD-tau-injected mice having significant tau pathology that were treated with IgG controls or either tau antibody (Fig. 8b). The contextual fear conditioning 24-h recall test demonstrated increased freezing behavior compared to that observed during training, demonstrating that all groups learned the contextual cues to anticipate foot shock (Fig. 8c). Upon remote recall testing 2 weeks after initial training, we did not observe any deficits in control IgG-treated AD-tau-injected 5xFAD mice with tau pathology compared to the PBS group lacking tau pathology. Thus, behavioral deficits were not detectable in the 5xFAD mice 3-months after AD-tau injection using these tests. Although this prevented assessment of whether SKT82 or DMR7 may provide cognitive benefits in this model system, the tau mAb treatments were well tolerated and did not independently elicit any behavioral deficits.

## Discussion

Here, we report two novel tau mAbs that bind selectively to the pathological misfolded conformation of tau compared to tau monomer, with discontinuous epitopes in the proline-rich central domain and the C-terminal domain of tau that are detectable in multiple human tauopathies. Binding of SKT82 and DMR7 to tau seeds prevents their uptake into primary neurons and inhibits the seeded aggregation of tau in WT mouse primary neurons and hippocampal slice cultures. Utilizing 5xFAD mice that harbor Aβ plaques, human brain-derived AD-tau injection induces robust endogenous mouse tau pathology that is inhibited by both SKT82 and DMR7, with SKT82 causing a reduction of both ipsilateral and contralateral tau pathology as assessed by IHC and biochemical extraction of insoluble tau from treated mice.

Conformation-selective tau mAbs have previously shown promise as immunotherapy candidates. TOMA antibodies selective for oligomeric tau species have been reported to reverse locomotor and memory deficits for 60 days following a single ICV or IV injection in aged P301L mice without influencing tau NFTs [39]. However, the relevance of these findings to human pathology are unclear, given the strong correlation of tau pathology to cognitive status in AD and the fact that the TOMA antibodies were raised against synthetic tau oligomers. In contrast, SKT82 and DMR7 were developed against human AD brain-derived insoluble tau extracts in order to target pathological tau species present in human disease. Passive immunotherapy studies with the conformation-selective MC1 antibody have demonstrated a reduction in hyperphosphorylated tau pathology but, like most tau mAb efficacy studies, these were evaluated in mouse models with overexpression of mutant human tau [36, 40, 41, 43], whereas DMR7 and SKT82 were evaluated in the context of physiological endogenous mouse tau with AD-tau seeded pathology. DMR7 and SKT82 were administered at a dose of 60 mg/kg by i.p. injection in this study which is consistent with previous studies [28, 41], although other tau mAbs have been tested at doses as low as 10 mg/kg [43].

In addition to conventional MC1 mAb administration, an AAV-based expression model of MC1 single chain variable fragment (scFv) has been examined in JNLP3 mice, which overexpress mutant human tau, resulting in reduced MC1-reactive tau, phosphorylated tau, and both soluble and insoluble total tau [61]. Passive immunotherapy approaches targeting pathological tau with a variety of linear-epitope and phospho-tau mAbs have also shown efficacy in multiple tauopathy mouse models [28, 30–38, 43], as have some active immunization approaches [62, 63]. This has led to the initiation of multiple clinical trials of both active and passive tau immunotherapy, although to our knowledge there is only one ongoing trial utilizing a conformation-selective tau mAb (clinicaltrials.gov NCT03518073) [44], which emphasizes the need for additional conformational-selective tau immunotherapy candidates.

Based on the comparable binding affinities, selectivity, and epitopes of DMR7 and SKT82, it was somewhat surprising that SKT82 was more effective than DMR7 at inhibiting tau pathology in slice cultures and the ipsilateral hippocampus in vivo*.* One possible explanation is that AD-tau bound by SKT82 may be cleared more actively since SKT82 is a mouse IgG2b isotype that activates microglial FC receptors, whereas DMR7 is mouse IgG1 that does not induce a microglial response in murine models [64]. Although we did not detect differences in microglial or astrocytic neuroinflammation in mice treated with SKT82 compared to DMR7 at the end of the study, there may be transient activation of clearance mechanisms induced by SKT82 (IgG2b isotype) at early time points following the initial intracerebral injection of AD-tau or more subtle phenotypic changes to microglia and astrocytes that are not apparent through IHC staining. The fact that both DMR7 and SKT82 showed a strong trend toward reducing tau pathology in the contralateral hippocampus might suggest that this isotype differences is not as critical to slowing the subsequent transmission of tau pathology to sites distant from the AD-tau injection site. Furthermore, the role of IgG Fc binding to mediate microglial clearance of tau aggregates is still unclear, as some groups report that the Fc domain is dispensable for mAb-mediated inhibition of tau pathology [61, 65], while other groups demonstrate Fcγ receptor binding and functional lysosomes are required for microglial-mediated clearance of tau aggregates [37, 66, 67].

Although there was high intergroup variability that reduced statistical power, DMR7 and SKT82 treatment of AD-tau injected 5xFAD mice nonetheless demonstrated similar efficacy to other tau-based passive immunotherapy approaches which generally result in a maximum of 50% reduction of AT8 pathological tau detected by IHC. The high-affinity mouse monoclonal antibody HJ8.5, from which the humanized variant that is in phase II clinical trials (ABBV8E12) was derived, showed similar variability and efficacy reducing AT8 positive tau pathology in 9-month old PS19 mice [28]. Treatment of 15-month old Tau784 mice with the pSer414-targeting tau monoclonal antibody also resulted in ~ 50% reduction in AT8-positive tau pathology by IHC [34] and similar results were observed in PS19 mice injected with tau MTBR PFFs and a pSer396 targeting tau mAb [33]. Furthermore, the MTBR targeting antibody DC8E8 resulted in highly variable and ~ 50% reduction of AT8-positive tau pathology in the R3m4 Tg mouse line [32]. Other groups have demonstrated a very acute reduction of AT8 pathology with a direct intracranial injection of a N-terminal tau antibody on the ipsilateral side of Thy22-tau Tg mice [26]. Yet other tau immunotherapy approaches have demonstrated no reduction in tau NFT-like pathology upon treatment with pan-tau antibodies and very modest reductions resulting from treatment with pSer404 antibodies in multiple transgenic human tau overexpression models [37].

Here, we demonstrate that DMR7 and SKT82 inhibit seeded aggregation of tau pathology in primary neurons by blocking the uptake of fluorescently-labeled tau seeds, consistent with mechanisms reported by others [19, 27, 32] . The epitopes of tau bound by mAbs may influence the ability of mAb to block neuronal uptake of tau [68]. We observed that binding of DMR7 and SKT82 to conformational epitopes containing the C-terminus and proline-rich domain provided greater inhibition of seeded tau aggregation in neurons than Tau5, which targets the proline-rich central domain of tau. One possibility is that the complex conformational epitope targeted by DMR7 and SKT82 may directly compete with, or sterically hinder, binding of tau to receptors such as low-density lipoprotein receptor-related protein 1 (LRP1), M1 and M3 muscarinic receptors, or heparan sulfate proteoglycans that have been proposed to mediate uptake of tau [69–71].

While most speculate that tau mAbs act to inhibit cell-to-cell transmission through sequestration of tau seeds in the extracellular space, there are reports that demonstrated the internalization of tau mAbs [38, 72, 73] and that internalized mAbs in complex with tau seeds bind to intracellular Trim21 receptors and are subsequently degraded [74]. The extent of tau mAb internalization may depend on the charge of the mAbs, which is an important consideration when humanizing mouse mAbs to maintain appropriate extracellular/intracellular distribution of mAbs to target the intended population of tau [75]. An intriguing alternative approach directly targets insoluble tau for intracellular degradation utilizing ubiquitin-targeting intrabodies [76], but much work remains to assess the efficacy, pharmacokinetics, and safety of such approaches.

We did not observe changes in the Aβ plaque burden upon induction of tau pathology with AD-tau injection or changes upon reduction of tau pathology with SKT82 or DMR7. This contrasts with prior reports of confounding tau immunotherapy influences on Aβ plaque load including reduction of Aβ plaques in 3x-Tg mice [29] and increased Aβ plaque load in Tg2576 mice [40]. Possible explanations for this difference may be the more aggressive plaque deposition in 5xFAD mice relative to 3xTg mice, or perhaps effects on plaques related to mutant tau overexpression in 3x-Tg mice that are not observed in our AD-tau-injected 5xFAD mice that express endogenous mouse tau. Our behavioral data for open field test and Y-maze are consistent with previous reports of this AD mouse model [60]. However, we did not observe changes in freezing during remote recall of contextual fear conditioning, which may be due to slightly younger mice used in our study or chronic handling of mice for weekly i.p. injections of mAbs [60].

## Conclusions

Given the great demand for tau immunotherapy candidates, it will be crucial to explore tau mAbs with varying affinities, epitopes, and isotypes. Here, we describe two novel candidates with unique conformational epitopes that selectively bind AD-tau compared to tau monomer. SKT82 and DMR7 reduce tau pathology in vitro and in an in vivo AD model with multiple neuropathological hallmarks of AD. Future studies to determine the effect(s) of mAb isotype in clearing tau pathology, and weighing any benefits against potentially deleterious neuroinflammation, will better define the optimal features of passive tau immunotherapy. As growing studies explore the complex interaction of Aβ and tau pathology, combination therapies targeting both tau and Aβ may also present an attractive approach.

## Supplementary information

**Additional file 1 : Figure S1.** Control IgG antibodies do not bind tau. Sandwich ELISA of CHL34 (IgG1 control) and 52S (IgG2b control) demonstrating lack of binding to tau monomer, AD-P1 PFFs, or AD-tau at the high concentration of 667 nM compared to active tau mAbs tested at 50–400 fold lower concentrations; Tau5 (8.9 nM), DMR7 (13.3 nM), and SKT82 (1.7 nM).

**Additional file 2 : Figure S2.** Tau mAbs do not influence amyloid-beta (Aβ) plaque burden in 5xFAD mice. X34 staining of Aβ plaques revealed no differences in the number or area of Aβ plaques between PBS- and AD-tau-injected mice. Furthermore, tau mAb treatment compared to IgG isotype controls did not alter the Aβ plaque load indicating that the reduction in tau pathology is not a result of altered Aβ levels. No statistically significant differences were detected across groups by one-way ANOVA with Tukey’s post-hoc analysis or unpaired t-test comparisons between treatment antibodies and the respective isotype control.

**Additional file 3 : Figure S3.** AD-tau and tau mAbs do not induce overt microglial changes. IHC staining of microglia with the Iba1 antibody demonstrates no apparent changes in microglial density or shape in the hippocampus and subiculum of AD-tau injected and tau mAb-treated mice compared to PBS injected mice lacking tau pathology.

**Additional file 4 : Figure S4.** AD-tau and tau mAbs do not induce astrocytic neuroinflammation. IHC staining of astrocytes with the GFAP antibody demonstrates no apparent changes in astrocyte density or morphology in the hippocampus and subiculum of AD-tau injected and tau mAb-treated mice compared to PBS injected mice lacking tau pathology.

## Data Availability

The datasets used and/or analyzed during the current study are available from the corresponding author on reasonable request.

## Abbreviations

AD: Alzheimer’s disease
mAbs: monoclonal antibodies
AD-tau: Alzheimer’s brain derived tau
Aβ: Amyloid beta
PFF: Preformed fibrils
Tg: Transgenic
WT: Wildtype
i.p.: intraperitoneal
nM: nanomolar
PHF: Paired helical filaments
NFT: Neurofibrillary tangles
NT: Neuropil threads
NP: Neuritic plaque
IHC: Immunohistochemistry
CBD: Corticobasal degeneration
PSP: Progressive supranuclear palsy
PiD: Pick’s disease
CSF: Cerebrospinal fluid
CNS: Central nervous system
ELISA: Enzyme linked immunosorbent assay
TBS: Tris buffered saline
IgG: Immunoglobulin
GFAP: Glial fibrillary acidic protein
Iba1: Ionized calcium binding adaptor molecule 1
