# Supplementary figures and images for: Conformation-selective tau monoclonal antibodies inhibit tau pathology in primary neurons and a mouse model of Alzheimer’s disease
Source: Mol Neurodegener. 2020 Nov 4;15:64. doi: 10.1186/s13024-020-00404-5 (PMC7643305; doi:10.1186/s13024-020-00404-5)

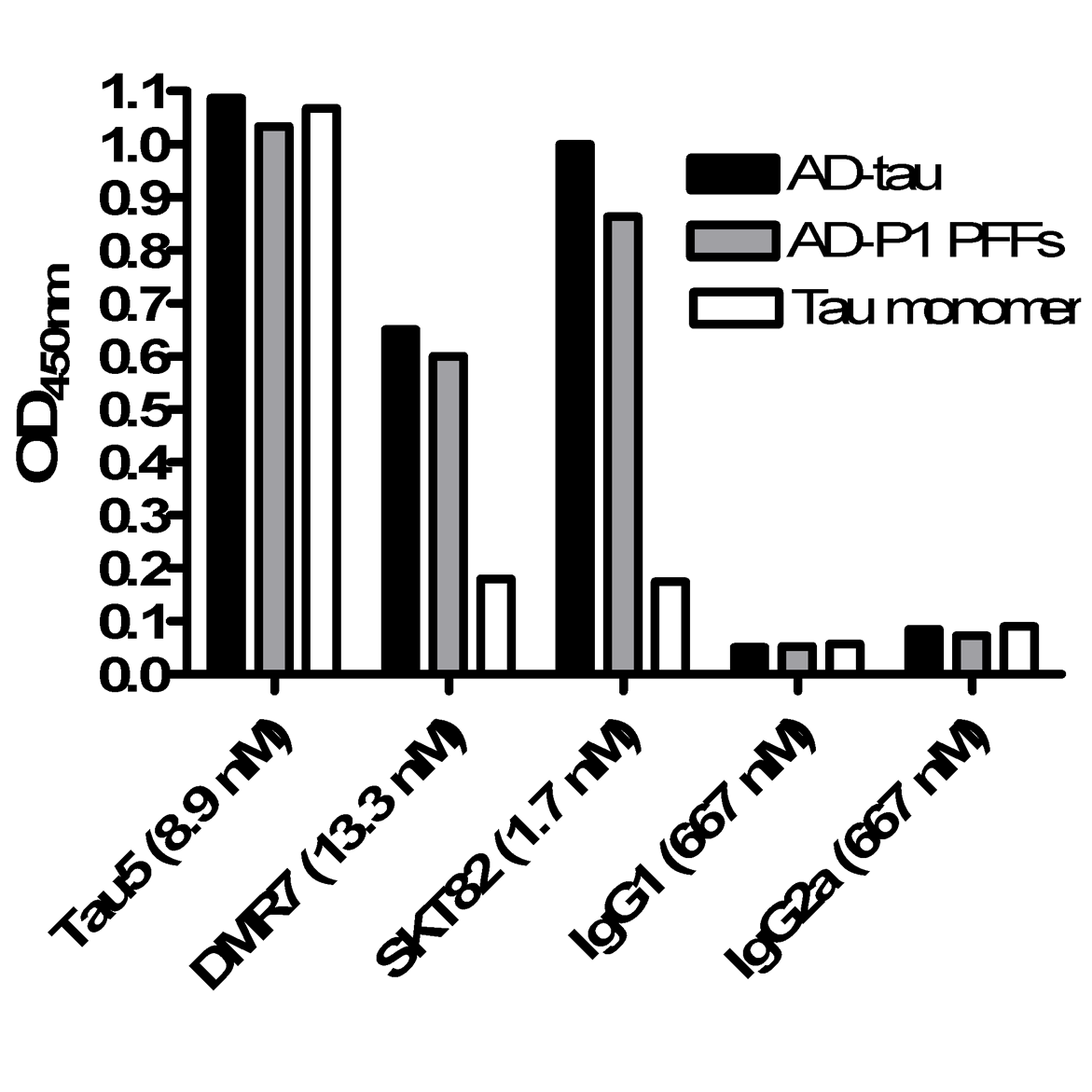

Supplement: Supplementary file 1 — Additional file 1 : Figure S1. Control IgG antibodies do not bind tau. Sandwich ELISA of CHL34 (IgG1 control) and 52S (IgG2b control) demonstrating lack of binding to tau monomer, AD-P1 PFFs, or AD-tau at the high concentration of 667 nM compared to active tau mAbs tested at 50–400 fold lower concentrations; Tau5 (8.9 nM), DMR7 (13.3 nM), and SKT82 (1.7 nM). [file 13024_2020_404_MOESM1_ESM.tif]

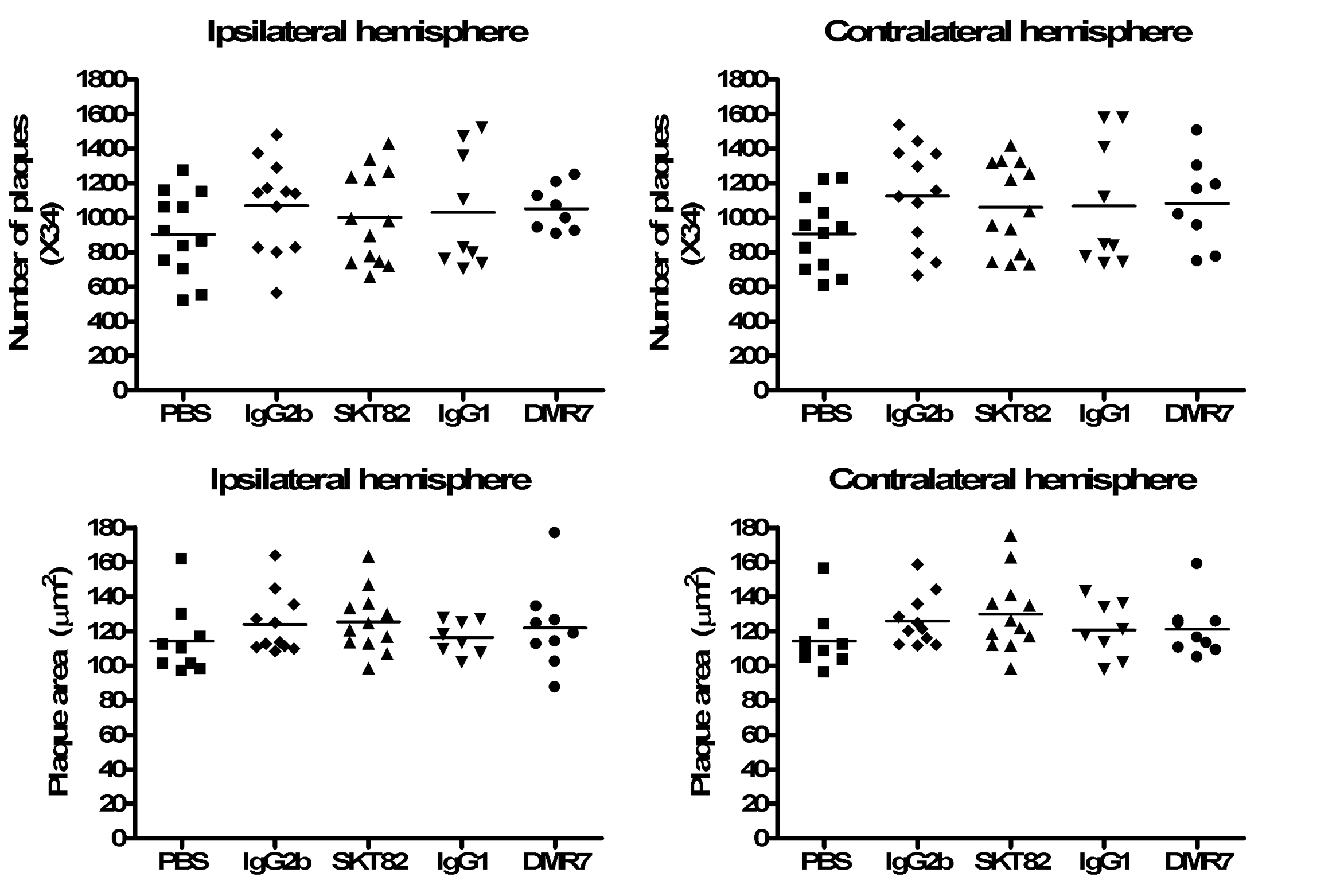

Supplement: Supplementary file 2 — Additional file 2 : Figure S2. Tau mAbs do not influence amyloid-beta (Aβ) plaque burden in 5xFAD mice. X34 staining of Aβ plaques revealed no differences in the number or area of Aβ plaques between PBS- and AD-tau-injected mice. Furthermore, tau mAb treatment compared to IgG isotype controls did not alter the Aβ plaque load indicating that the reduction in tau pathology is not a result of altered Aβ levels. No statistically significant differences were detected across groups by one-way ANOVA with Tukey’s post-hoc analysis or unpaired t-test comparisons between treatment antibodies and the respective isotype control. [file 13024_2020_404_MOESM2_ESM.tif]

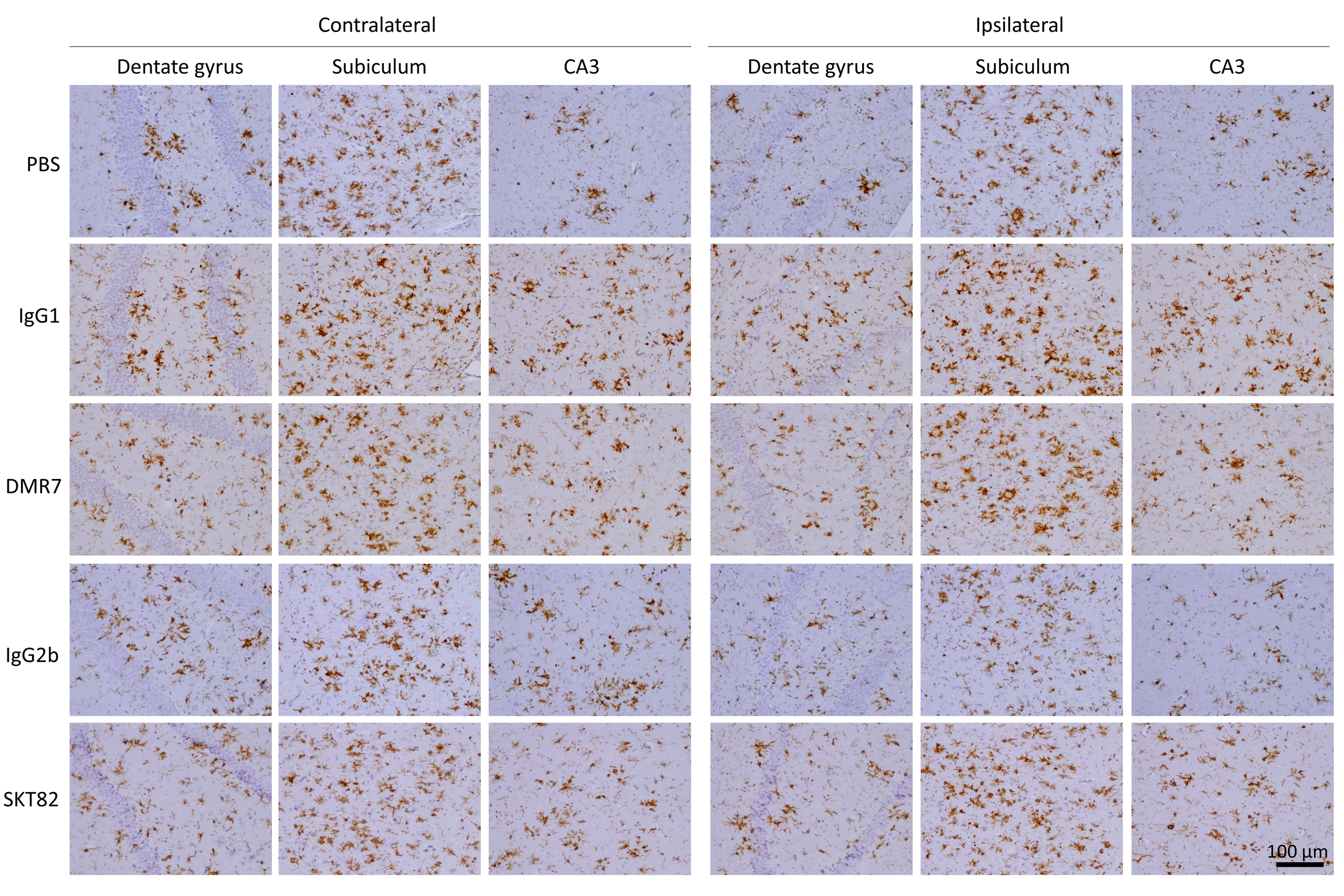

Supplement: Supplementary file 3 — Additional file 3 : Figure S3. AD-tau and tau mAbs do not induce overt microglial changes. IHC staining of microglia with the Iba1 antibody demonstrates no apparent changes in microglial density or shape in the hippocampus and subiculum of AD-tau injected and tau mAb-treated mice compared to PBS injected mice lacking tau pathology. [file 13024_2020_404_MOESM3_ESM.tif]

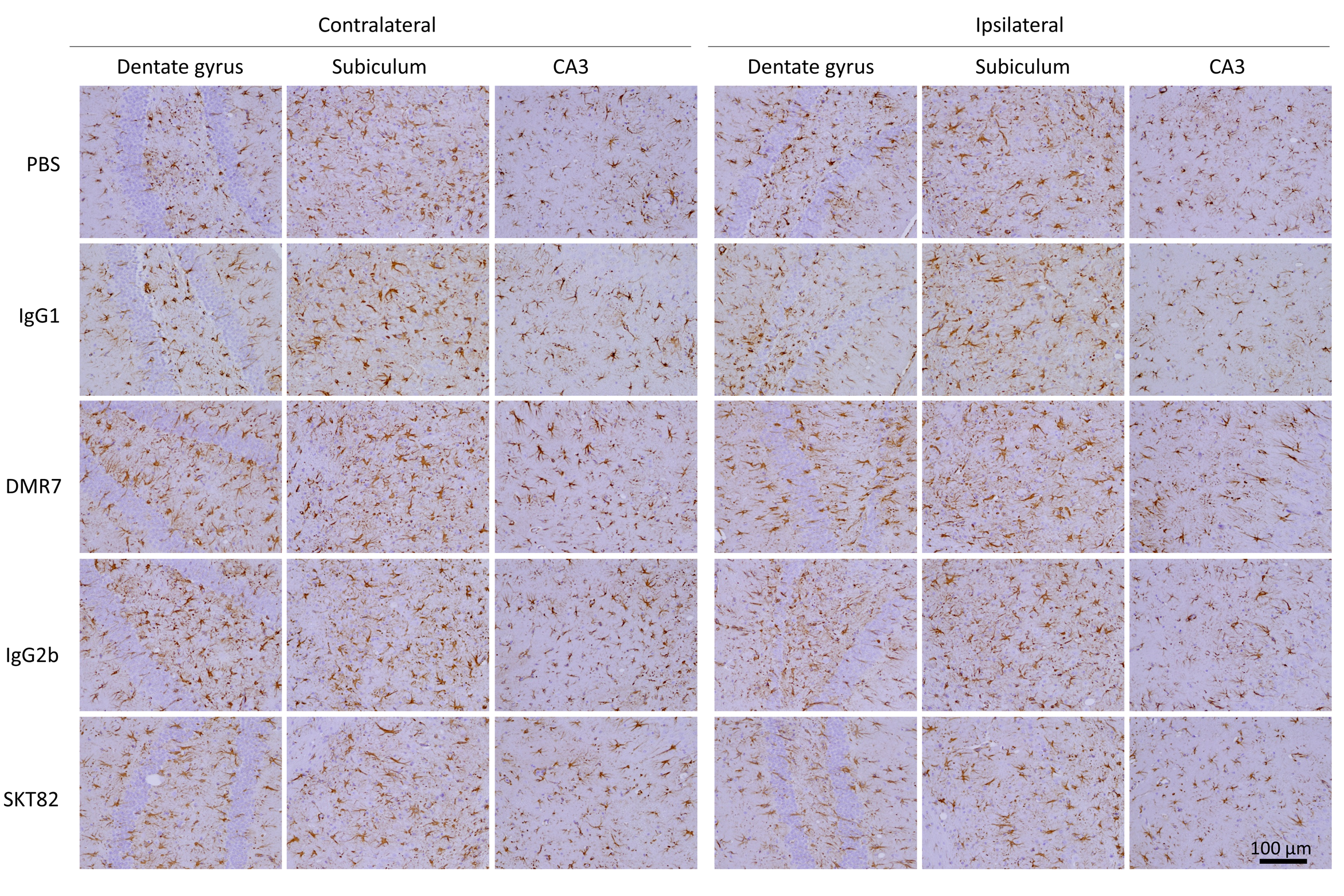

Supplement: Supplementary file 4 — Additional file 4 : Figure S4. AD-tau and tau mAbs do not induce astrocytic neuroinflammation. IHC staining of astrocytes with the GFAP antibody demonstrates no apparent changes in astrocyte density or morphology in the hippocampus and subiculum of AD-tau injected and tau mAb-treated mice compared to PBS injected mice lacking tau pathology. [file 13024_2020_404_MOESM4_ESM.tif]
